# Imagining Interventions for Collective Sex Environments

**DOI:** 10.1007/s10508-018-1222-7

**Published:** 2018-05-10

**Authors:** Paul Flowers, Jamie Frankis

**Affiliations:** 1 M420, Department of Psychology, George Moore Building, Glasgow Caledonian University, Glasgow, G4 0BA Scotland, UK; 2 Department of Nursing, Glasgow Caledonian University, Glasgow, Scotland, UK

Frank’s (2018) article provides an excellent summary of transdisciplinary research concerned with collective sex environments, synthesizing highly diverse studies spanning five decades. The contributing papers utilize a broad range of methods and reflect many key sexual health risks across several diverse and distinct populations. For many readers, such as ourselves, with a particular interest in a single population, Frank’s synthesis provides a much needed and entirely fascinating wider perspective. This overarching vantage point can teach us about similarities and differences across populations, while simultaneously illuminating the populations and research we know so well through a different lens. As such, the paper provides an essential contribution to the literature. However, rather than champion the paper’s many strengths, within this Commentary we wish to grapple with what could be seen as its potential shortcomings. Our aim here is not be critical for the sake of it, but to somewhat playfully push debates further about many issues addressed within the paper. In this way, we wish to initiate more dialogue concerning collective sex, concomitant risks, and imaginative ways to ameliorate such risks.

## Looking Backwards Not Looking Forwards

First and foremost among the “shortcomings” of Frank’s (2018) article is the retrospective gaze of the review methodology. Of course, this limitation is not unique to this article and represents a potential failing for all review work. However, the necessary retrospective focus of a review, the demands of synthesis, and the need to build cumulative knowledge though layering new information upon what has gone before brings multiple challenges.

Critically, review epistemology in general fails to accommodate recent and contemporary changes and cannot address the anticipation of the future. Adopting a retrograde and consolidating gaze also tends to focus upon the common denominators across previous research. This drags the focus of cumulative knowledge retrospectively and is ill-equipped to grapple with major and more recent change.

For us, this epistemological limitation is central to explaining the lack of critical mass identified within the review that is concerned with what could be called the democratization of collective sex. We would suggest that the recent trends in democratizing collective sex have materialized through the world of social media and the proliferation of dating/sexual hookup smartphone apps, and their associated Web sites (Davis, Flowers, Lorimer, Oakland, & Frankis, 2016; Wu & Ward, 2018). Wider innovations within sexual health also shake the simple sedimentation of transferable knowledge across the period of time encompassed by the review. For example, in relation to HIV, biomedical innovations such as pre-exposure prophylaxis or wider treatment as prevention have profoundly changed the somatic effects of condomless sex for many populations around the world (Frankis, Young, Lorimer, Davis, & Flowers, 2016; Young, Flowers, & McDaid, 2015, 2016). Equally, in relation to antimicrobial resistance, the prevalence of drug resistant gonorrhea has also changed dramatically across the last half century reintroducing risks to “safer” sexual acts (Unemo & Nicholas, 2012). Among those who use collective sex environments, an understanding of these issues changes the choreography of sexual conduct across time. Along these lines, the risks associated with condomless anal sex are very different now than they were within the recent past encompassed within the review (Jin et al., 2015).

We also have frustrations that relate to a tendency within the review to “short change” the reader in relation to conceptual clarity. Arguably, these represent key lost opportunities for synthesis within the review. To some extent, the messy nature of transdisciplinary work and paradigm-specific thinking reverberates throughout the review, rather than there being a sense of attempted cohesion. Of course, to these ends we have tremendous sympathy with Frank; the unthankful task of the reviewer is to enter the mire of other people’s thinking and, given the heterogeneity of the research included, other people’s disciplines. To expect an author to untangle, dismantle, and then rebuild and explain this complexity is certainly a tall order but one which, if successful, could have added much to the field. There is a sense that stopping short of delivering conceptual clarity within the paper represents a key device that enables many of the review’s central arguments to develop. These issues are explored within the sections below.

## Toward a Typology of Collective Sex Environments

As Frank (2018) notes within the paper, harmonizing nomenclature is particularly challenging given the diverse ways various authors have attempted to classify the complexities of collective sex environments. Yet, given the time and energy put into the review, it is a shame that there was a lack of categorization or visualization, to map out major patterns within the heterogeneity of the review literature. For us, this represents a lost opportunity to synthesize and then articulate how sex researchers *should* communicate about collective sex environments from this point forwards.

We believe Frank’s review work represents a chance to lead the interdisciplinary field in distinguishing and discussing the range of collective sex environments (e.g., public sex environments, saunas, gyms, erotic oases, public sex venues, bathhouses, sex clubs, group sex events, lifestyle events, lifestyle groups, group sex, public sex, sex parties, chem-sex) and furthermore the kinds of people who use them. Indeed, such an endeavor would be invaluable. With an agreed framework and vocabulary, it becomes possible to shape the collection and synthesis of evidence about the effectiveness of various interventions and their active components within specific contexts and specific populations. However, Frank does not provide a typology of collective sex environments (i.e., detailing their similarities and differences). In contrast, she emphasizes the plurality and specificities of a range of environments used for collective sex. This necessarily adds weight to the paper’s general argument that attending to such particularity is pointless. In contrast, we believe that ordering the potential chaos of such pluralism and examining the patterning of shared and distinct elements of collective sex environments could represent a viable and useful focus for review. Building such firm foundations would offer a step forward in understanding the contributions of previous intervention evaluations across collective sex environments. In turn, this would enable us to develop improved and more effective interventions which mitigate the range of potential risks that are related to collective sex–above and beyond those concerned with sexual ill-health alone.

## Toward Conceptual Clarity Regarding Theory and Theoretical Lenses

In a parallel critique to that outlined above, we also believe this review represents a lost opportunity with regard to developing and synthesizing theory. As Frank (2018) notes, the way theory is used across the literature is again highly diverse and reflects a number of distinct and potentially incommensurate paradigms. Given the heterogeneity of the work covered within the review, we fully acknowledge that there are major challenges in understanding and integrating multi-leveled and diverse theoretical approaches and their attendant theoretical constructs. Indeed, a range of specified and unspecified theoretical constructs are at play within this literature and arguably, this plurality of theory has limited the field. Some explanatory theory is heuristic for example; in contrast, some attempt to present a veridical model of sexual conduct. Other theory still is pitched at levels which are far more macro and relate to different ontologies, while some researchers adopt more meso-social or micro-social theories; all of which complicates synthesis still further.

Frank notes, for example, that the literature draws upon literary and critical theory, feminist theory, queer theory, Marxist theory, materialist theory, psychological theory, anthropological theory, theories of risk, sexual cultures and socio-cognitive theories. She also highlights the overarching influence of symbolic interactionism. Once more, given the heterogeneity of theory within the contributing literature, it could be argued that this review represents a further lost opportunity to synthesize the theoretical positioning and illuminations of the work covered therein. Mapping how theories and their theoretical constructs relate to each other, then detailing how this explains specific aspects of the social organization of collective sex would deliver even further added value. Admittedly, the ontological assumptions underpinning certain theories used within the contributing review studies ensure this would be no easy or straightforward task. Yet to come so close to synthesizing this literature in this way and not quite delivering has left us wanting that little bit more from the review.

Meta-theoretical perspectives, such as the socio-ecological model (Baral, Logie, Grosso, Wirtz, & Beyrer, 2013; Bronfenbrenner, 1979), may have been useful in organizing some of the maelstrom of theoretical perspectives and constructs, which left unorganized occlude a theoretical appreciation of collective sexual conduct. Nevertheless, Frank’s paper does not organize the theoretical contributions across the field. It does not explain the unique contribution associated with each theoretical perspective, nor does it illuminate the complementary and potential synergistic effects of diverse theoretical lenses. Instead, the interrogation of theory tends to be used rhetorically, to add momentum to the paper’s overall argument. This overall argument suggests that theory, or the specification of theoretical constructs, has gone *too far* in relation to the particularities of sexual conduct within particular kinds of collective sexual settings. By staging the complexity of theory in this way, the paper builds toward an argument that simpler theory is warranted and for Frank these theories of choice appear to be concerned primarily with deviance and transgression.

## Paradigms and Pathology: Transgression and Deviance

The selection and priming of theory in relation to the adoption of the anthropological framework of transgression and its resonance with deviance is a little troubling for us. Firstly, because these concepts are rooted within pathology and secondly, we are not convinced collective sex is *not* normative.

In relation to the orientation of transgression and deviance to pathology, Frank (2018) claims to *not* examine deviant people, places and practices. However, it is unclear to us how the theoretical lenses of transgression and deviance *cannot* necessarily illuminate their subjects in these particular ways and, specifically, through a pathologizing lens. This particular conceptual armament positions deviants as agentic perpetrators of transgression and simultaneously underemphasizes the social organization of sex in relation to variations in time and place. For gay and other men who have sex with men, for example, historically toxic hetero-normative social structures and associated infrastructure have constrained the possibilities of sex in ways which were not entwined with collective sex.

In countries and in eras where men could not, or cannot, cohabit as couples, or meet within safe, legal infrastructures of commercial gay venues, then the only means to access other men for sex was, and is, within public sex venues (Chauncey, 2014; Santos, Makofane, Arreola, Do, & Ayala, 2017). In turn, these constraints shape sexual conduct and contribute to such places as being risky, primarily because of the threat of homophobic violence and the concomitant risks of social exposure within a hetero-normative social world. Similarly, there are profound differences between the social organization of collective sex in the past and the present (Berlant & Warner, 1998; Prior & Hubbard, 2017; Wu & Ward, 2018). Historically, the social organization of sexual opportunities between men also shaped their sexual interactions, driving men to anonymous sex environments and limiting the social repercussions of such sexual interactions, for example, curtailing easy access to the development of longer-term relationships and romantic intimacy. Once herded into such narrow spatially defined places, heightened vulnerabilities for homophobic violence and stigma emerged. Thankfully, in some places around the world, this historic funneling of opportunities for sexual connection between men into narrow collective spaces has changed dramatically across recent decades in many places across the globe, with equality and legislation creating new possibilities of being for men seeking sex with other men. In some cities, there has been a proliferation of venues profiting from the commercialization and commodification of collective sex, and in cities with large enough populations to support niche markets, this has enabled particular venues to specialize in particular *kinds* of collective sex. In this way across the scope of Frank’s review, there is a sense that some men in the past had no choice but to engage in collective sex yet now, in contrast, there are many choice-based opportunities to engage in varied kinds of collective sex.

More recently, the explosion of geo-spatial sexual networking has opened up a proliferation of virtual and real spaces, in which (collective) sex can be negotiated and realized. This has transformed traditional public and commercial sexual spaces but, critically, also created new possibilities around “pop-up” or opportunistic sex parties in private homes, often advertised via GPS sexual networking as only semiprivate or even public events. As such, seismic changes have taken place in relation to the drivers of collective sex in many places across the world. Much of the literature cited within Frank’s article the paper is old and inevitably somewhat dated. In particular, this contributing literature cannot address the major impacts GPS-based technology and digitally realized sociosexual networking has had in relation to changing the drivers of, and creating new facilitators for, collective sex. Therefore, where GPS-based technology is currently available there is a clear sense of the amplification of agency and choices associated with collective sex, which did not exist in the past.

Although the digitization of sexual opportunities is mentioned within the review, for example in relation to the introduction of GPS-based mobile applications, we believe this actually represents a critically transformative moment delivering profound cultural change and major transformations within sexual cultures for very large sections of the population. Within Frank’s paper, there is an absence of any single definition of what constitutes a collective sex environment. But for us, the increasingly public world of smartphone dating apps facilitating sexual conduct shares far more in common with the legacies of many collective sex environments than it does with the social organization of sex *per se* across recent millennia. In this way, one could argue that there has been a democratization of collective sex, with large segments of the population engaging collectively through dating apps with the shared and public commodification of both the sexualized self and the sexualized other. These platforms clearly share core elements with other collective sex environments including features securing site users’ physical, social and psychological safety. Like many collective sex environments, space and time are segmented to enable site users to progressively enter into more explicit interactions, and there are clear social norms focused upon ascertaining intentions, preferences and consent. Moreover, much dating app architecture is concerned with gate-keeping functions, with demarcations of public and more socially oriented space (e.g., profile information or discursive spaces) versus those that are more private and directly concerned with sexual negotiation and interaction. Along these lines, it is increasingly hard to engage with collective sex environments as either transgressive or as deviant; indeed, they appear to be increasingly normative. In relation to imagining interventions for health and well-being in the future for those who use such dating apps, much could be learned from the long and interesting history of collective sex environments as detailed within the paper.

## Toward Theory-Informed Interventions

We would also argue that the failure of Frank (2018) to coherently organize, map, and sift diverse theory into some coherent order limits the ways we can imagine interventions to improve health and well-being and reduce diverse risks associated with collective sex. With regard to theorizing interventions, there is little attempt within the review to understand how the existing literature can offer a wide variety of complementary and socially nuanced, theory-informed interventions. Sadly, it could be argued that the paper offers little sense of clarity, or even hope, for future interventions and, arguably, elicits a sense of pessimism and premature foreclosure with regard to intervention possibilities overall. For example, toward the end of Section I much is said about the apparent impossibility of understanding the heterogeneity of people and places in relation to focusing intervention efforts. Yet, such inertia can easily be overcome with a sense of how interventions and their content can be targeted and tailored to meet the specific needs of particular users at particular times in particular places. In this way, and especially in light of digitally delivered technologies where they are available, there is ample opportunity to address the “tendency to particularity” with a range of bespoke interventions, with targeted and tailored active content, that reduce risks and improve health and well-being.

Equally, within the paper, far more could be made of examining the available literature from an explicitly salutogenic perspective. Such an a priori focus could identify and detail the successful components of self-regulating interventions that already preserve the health and well-being of those who use collective sex environments. Collating such evidence can rapidly produce a blueprint of potentially culturally appropriate intervention elements that can be transferred to existing and emerging collective sex environments, or deployed as rapid responses to infectious disease outbreaks or other, more social, harms within particular spaces. Identifying beneficial intervention components has a clear resonance with meso- and micro-social theory and related theoretical constructs. Potentially transferable, “home-grown” intervention elements are mentioned within Frank’s paper. For example, this includes the importance of creatively attaining consent either nonverbally or preceding sexual conduct, and imaginative means of clarifying and communicating the norms and collective expectations of conduct. Alternatively, within the review, there is a clear sense of how intervention elements associated with both identities and communities figure as important protective issues therein, as do amplified feelings of responsibility for the generic other(s) who share those collective sex environments. These dynamics represent gold dust for co-designed and culturally appropriate intervention content for increasing health and well-being associated with collective sex environments.

We would suggest that embracing the logic of co-produced interventions, or seeking to understand the self-organizing and self-regulating harm reduction elements of collective sex environments, jars with Frank’s thinking concerning how and why interventions are developed and deployed. Within her section “Not the Time or the Place,” there is an odd, and we would suggest rather unproductive, sense of “*them”* and “*us*.” Frank’s emerging argument, that interventions should not be delivered *in situ,* is built here upon a falsely dichotomous foundation. Her dichotomy suggests that there are two groups of people: those who use collective sex environments and those who seek to impose interventions upon those users. Setting up such a binary imbues readings of such situations with power and conflict. Moreover, it diminishes the appreciation of the self-organizing, harm-reducing, health protective assets that have developed within many communities who use collective sex environments. Furthermore, it strips away or overlooks the agency and care of those who use collective sex environments and positions them as both deviant and having particular deficits. Rather, we argue that empirically and theoretically informed, ideally co-produced interventions could replace this negative dichotomy with the possibility of positive, community-led social change and health enhancements.

Finally, we argue that a pluralistic understanding of theory, pitched at various complementary levels, is vital to deliver the best kinds of interventions. For example, we need evidence driven theories that explain what happens within the individual, between individuals, within communities, within societies and across cultures, in order to design and deliver the best, theoretically informed interventions to improve health in the widest sense. In this way, we agree wholeheartedly with Frank that it is vital to imagine interventions that are directly concerned with changing social structures, including, for example, legislation and policies that directly address inequalities, social marginalization, poverty, and associated reduced educational opportunities. While such interventions make the most of macro-social theory, we believe, however, that it is equally important to imagine theory-based interventions that function at the meso- and micro-social levels. Although Frank’s review provides a space for this dialogue to begin, our aim herein has been to highlight some of the critical issues that still remain unaddressed, that can be translated into social action and risk reduction. These kinds of interventions can deliver holistic health benefits.

To summarize, we believe Frank’s review to be an excellent contribution to the literature yet we believe there is still much to be learned from the historical evidence around collective sex environments. There is still more that could be offered from a synthesis which drives forward theoretical and empirical development, redresses the narrative of deviance and pathology, postulates their increasingly normative nature and, critically, advances the impact of digital networking technologies therein toward health enhancements.
